# Lameness Affects Cow Feeding But Not Rumination Behavior as Characterized from Sensor Data

**DOI:** 10.3389/fvets.2016.00037

**Published:** 2016-05-10

**Authors:** Vivi M. Thorup, Birte L. Nielsen, Pierre-Emmanuel Robert, Sylvie Giger-Reverdin, Jakub Konka, Craig Michie, Nicolas C. Friggens

**Affiliations:** ^1^INRA, AgroParisTech, Université Paris-Saclay, UMR 791 Modélisation Systémique Appliquée aux Ruminants, Paris, France; ^2^NBO, INRA, Université Paris-Saclay, Jouy-en-Josas, France; ^3^Department of Electronic and Electrical Engineering, University of Strathclyde, Glasgow, UK; ^4^Silent Herdsman Ltd., Glasgow, UK

**Keywords:** accelerometer, automation, behavior, lameness, animal, phenotyping, dairy cattle

## Abstract

Using automatic sensor data, this is the first study to characterize individual cow feeding and rumination behavior simultaneously as affected by lameness. A group of mixed-parity, lactating Holstein cows were loose-housed with free access to 24 cubicles and 12 automatic feed stations. Cows were milked three times/day. Fresh feed was delivered once daily. During 24 days with effectively 22 days of data, 13,908 feed station visits and 7,697 rumination events obtained from neck-mounted accelerometers on 16 cows were analyzed. During the same period, cows were locomotion scored on four occasions and categorized as lame (*n* = 9) or not lame (*n* = 7) throughout the study. Rumination time, number of rumination events, feeding time, feeding frequency, feeding rate, feed intake, and milk yield were calculated per day, and coefficients of variation were used to estimate variation between and within cows. Based on daily sums, using each characteristic as response, the effects of lameness and stage of lactation were tested in a mixed model. With rumination time as response, each of the four feeding characteristics, milk yield, and lameness were tested in a second mixed model. On a visit basis, effects of feeding duration, lameness, and milk yield on feed intake were tested in a third mixed model. Overall, intra-individual variation was <15% and inter-individual variation was up to 50%. Lameness introduced more inter-individual variation in feeding characteristics (26–50%) compared to non-lame cows (17–29%). Lameness decreased daily feeding time and daily feeding frequency, but increased daily feeding rate. Interestingly, lameness did not affect daily rumination behaviors, fresh matter intake, or milk yield. On a visit basis, a high feeding rate was associated with a higher feed intake, a relationship that was exacerbated in the lame cows. In conclusion, cows can be characterized in particular by their feeding behavior, and lame cows differ from their non-lame pen-mates in terms of fewer feed station visits, faster eating, less time spent feeding, and more variable feeding behavior. Further, daily rumination time was slightly negatively associated with feeding rate, a relationship which calls for more research to quantify rumination efficiency relative to feeding rate.

## Introduction

Disease incidence during early lactation is substantial in dairy cows (1). Automated monitoring can replace subjective and time-consuming visual observations and provide early identification and facilitate provision of treatment of vulnerable animals, which in itself is valuable. Additionally, precision livestock farming (PLF) technologies (2) such as accelerometers that provide individual time-series of activity present a unique opportunity to refine the phenotypic characterization of dairy cows. For instance, the variability associated with time-series data from PLF technologies can be used to estimate a complex phenotypic trait like robustness, see Ref. (3), which will be valuable for individually targeted management, disease prevention, and not least genetic selection. Currently, the utilization of PLF technologies for phenotyping is largely unexploited (4).

Most diseases affect the feeding and rumination behavior of the cow, and changes in feeding and rumination are key behavioral indicators of compromised health in ruminants. An example of this is the decrease in rumination and feeding time of cows following subjection to a mastitis challenge (5). Rumination time and feeding rate decreased, whereas feeding time increased in cannulated cows the day after being subjected to a ruminal acidosis challenge (6). In goats, a feeding pattern of few, long meals (vs. more frequent and shorter meals) has been linked with a lower rumen pH, which may increase the risk of rumen acidosis (7). Lameness has been shown to affect a number of behavior characteristics, in terms of decreased daily feed intake, feeding time (8–10), decreased rumination time (8, 11), and increased feeding rate (9, 10). However, none of these studies investigated lameness, rumination, and feeding behavior at the same time.

Other factors, such as lactation stage, management, feed composition, and environment, influence feeding and rumination behaviors. During early lactation, feed intake increases (12), and cows have been found to exhibit rumination peaks 1–2 h after feeding peaks (13). Moreover, decreasing forage particle size decreased daily feeding and rumination time (14). Not least, cows are gregarious animals that often synchronize behavior; therefore, their social and physical environment may impose constraints on their feeding and rumination (15). It is important to take these influential factors into account when studying changes to feeding and rumination in relation to lameness.

Automated technologies that measure in real time can detect short-term behavioral changes, such as decreases in feed intake, feeding rate, and feeding and rumination time around estrus (16). Moreover, lameness has been found to decrease night-time rumination (17). To the best of our knowledge, the effect of lameness on feeding and rumination behavior measured simultaneously using PLF technologies has not yet been studied. This study aimed to characterize the individual feeding and ruminating behavior of dairy cows based on data from automated feed stations and neck-mounted accelerometers measuring movements over time in three dimensions, and to quantify the effect of lameness on feeding and rumination.

## Materials and Methods

### Animals and Housing

The experiment was carried out at the research farm of Scotland’s Rural College (Crichton Royal Farm, Dumfries, UK), 20 lactating Holstein Friesian cows were loose housed in one pen. The pen had 24 cubicles with rubber mattresses, top-dressed with saw dust, and grooved concrete floors in the alleys with automatic scrapers. Cows were milked three times daily in a milking parlor: the milking periods ranged from 0730 to 1000 h; 1430 to 1630 h; and 2130 to 2330 h. The cows left the pen as a group for about 40 min/milking. In the milking parlor, the cows were offered 0.6 kg concentrate/day spread over the three milkings. In addition, high yielding cows were offered 1 kg/day concentrate extra per 3 l milk yield above 30 l/day, i.e., a cow yielding 36 l milk/day would receive 2.6 kg concentrate/day in the parlor. The feed offered during milking is not included in the analysis reported here, and only one cow (which was not lame) did consistently yield >30 l/day during the experiment. Cows had *ad libitum* access to water from two water troughs, one at each end of the pen. Water intake was not recorded. The experiment started on June 23, 2014, hereafter referred to as day 1, and lasted for 24 days. The cows were permanently in the pen from day 6 and throughout the experimental period, except for short periods during normal farm routines such as milking and pregnancy detection.

All 20 cows were equipped with neck-mounted accelerometers. However, two cows past 22 months in lactation were excluded from analysis, and accelerometer data could not be retrieved from two cows, leaving 16 cows in the final data set used for analysis. Two cows were in their first lactation, six cows were in their second lactation, and eight cows were in their third lactation or more (mean number of days in milk was 238 ± 91.1 days). At trial end, 8 of the 16 cows were pregnant (mean number of days in gestation was 137 ± 80.2 days). Milk yield from the three daily milkings was obtained for 285 cow days (out of 311) for which mean milk yield was 22.4 ± 7.13 l/day.

### Locomotion Scoring

The cows were locomotion scored by one of two trained and highly experienced herdsmen on days 1, 6, 12, and 22 when cows exited the milking parlor. These herdsmen perform locomotion scoring on 250 cows/week and 52 weeks/year on alternate weeks. The herdsman performing almost all of the assessments in this study has locomotion scored the cows at Crichton Royal Farm for 15 years. The herdsmen have regular calibration sessions with the farm veterinarian to minimize observer drift between them. The scoring method has been modified from Ref. (18) and can be described as follows: (1) perfect even tracking (tracking: the hind foot is placed on the same spot on which the fore foot was placed) and no adduction or abduction (abduction/adduction: the cow swings its hind legs inside or outside to achieve tracking); (2) adduction or abduction, but normal tracking or not tracking; (3) slight lameness with uneven or short strides; (4) obvious lameness with difficulty in turning; (5) severe lameness with difficulty in walking. All cows were either consistently not lame (score <3) or consistently lame (score ≥3) throughout the study, with the exception of one cow, which was a score 4 until treatment for digital dermatitis 12 days from the end of the study. We categorized this cow as belonging to the lame group. According to this lameness categorization (not lame vs. lame), seven cows were not lame, and nine cows were lame throughout the experiment.

### Feed and Feed Stations

Cows were fed a total mixed ration (TMR), which was a mixture of grass silage, concentrates in pellets, wheat, wheat straw, and additives with an average of 387 g dry matter (DM)/kg feed, 344 g neutral detergent fiber (NDF)/kg DM, 185 g crude protein/kg DM, 43.3 g fat/kg DM, and a metabolizable energy content of 11.8 mj/kg DM. The TMR was distributed in 12 automated feed stations (Roughage Intake Control System, Insentec BV, Marknesse, The Netherlands) recording weight to the nearest 0.1 kg. There were barriers on the sides of each feed station to minimize displacements and stealing. The use of these feed stations for characterizing feeding behavior has been described earlier (19). All cows had access to all feed stations. A preliminary Wilcoxon test of the lame group vs. the not lame group showed that lameness did not affect how cows distributed their feed intake between feed stations. Fresh TMR was distributed once daily before noon. Fresh matter intake (FMI) was recorded around the clock, except from 1,145 to 1,215 h due to a feed station resetting procedure, yet cows had access to the feed stations at all times. By examining the differences in feed bin content before and after the resetting procedure, we calculated that a daily average of 2.8 kg fresh matter (FM)/feed station (range from 0 to 11.4 kg) was eaten without being assigned to cows. Per cow, this corresponds to 1.7 kg/day of unaccounted FMI. Given a feeding rate of 250 g/min, this amount would take less than 7 min to eat. For historical reasons, a maximum FMI of 5 kg/visit was allowed, meaning that at 5 kg, the feed station door went up, thus forcing the cow to withdraw from the feed station. However, cows were able to override the limit by keeping the door down. Feeding time was assumed to be equal to visit duration even though the cow may not have been actively ingesting feed during the entire visit.

### Feed Data Cleaning

Visits with a duration of 0 s (145 out of 14,053 visits) were excluded. For each individual cow, FMI was regressed on visit duration with the regression line passing through the origin. A double check similar, but not identical to the one described by Bossen et al. (20) was performed. In a first check, outliers were identified as (1) visits deviating more than ±5 SD units from the first regression line, and (2) visits with both a duration <4 s and FMI >0.1 kg. The outliers were set to missing, a second regression was performed, and the missing values were replaced by values calculated from the coefficients of the second regression. In a second check, visits deviating more than ±5 SD units from the second regression line were immediately replaced by values calculated from the coefficients of the second regression. For visits with a large or negative FMI, FMI was replaced. For long visits with a low FMI, duration was replaced. For visits <4 s with FMI >0.1 kg, duration was replaced. Out of 13,908 feed station visits, 3.4% of the visits were affected by these data checks: 264 FMI and 202 durations.

### Accelerometers and Rumination Classification

The cows were equipped with a neck-collar fitted with an accelerometer (Silent Herdsman Ltd., Glasgow, UK) sampling in three dimensions at 12 Hz. The accelerometers were a prototype; therefore, data were stored on the SD-card of the accelerometer and transferred manually to a local computer when the accelerometers were taken off. To ensure data storage, two batches of accelerometers were used in serial. The first batch collected data from day 1 to 9 and the second batch collected data from day 10 to 25. Because days 9 and 10 were not full days of data collection, they were removed from the data set. Unfortunately, data from four accelerometers were corrupted either in the beginning or in the end of the experimental period. Therefore, the accelerometer data consisted of time-series from 16 cows with on average 19.4 full days of data (ranging from 8 to 22 days), in total 311 cow days.

A combination of the estimated variance in the accelerometer signal (an expression of energy content) and the frequency content derived from the Fourier transformed accelerometer signal were used for classification of rumination (21). The classification was validated by use of a RumiWatch halter (Itin ± Hoch GmbH, Liestal, Switzerland), which detects rumination *via* a pressure sensor (22). Using Hidden Markow Models, rumination was classified with a sensitivity of 86.1% and a positive predictive value of 98.7% (21). There were 7,697 events of rumination during the 311 cow days. All rumination and feeding characteristics used for analysis are described in Table 1.

**Table 1:** **Descriptions, units, number of records, and abbreviations used in text for all behavior and milk yield characteristics**.

Description	Unit	*n*	Abbreviation
Milk yield	l/day	285	dMY
FMI per visit	kg/visit	13,908	vFMI
Feeding duration per visit	s/visit	13,908	vFdur
FMI (fresh matter intake)	kg/day	311	dFMI
Feeding time	min/day	311	dFtime
Feeding frequency	visits/day	311	dFfreq
Mean FMI per visit	kg/visit	311	mvFMI
Mean feeding duration per visit	s/visit	311	mvFdur
Daily feeding rate	g/min	311	dFR
Cow feeding rate	g/min	16	cFR
Rumination duration per event	min/event	7,697	eRUMdur
Rumination time	min/day	311	dRUMtime
Rumination frequency	events/day	311	dRUMfreq
Mean rumination duration	min/event	311	meRUMdur

### Statistical Analysis

All analysis was performed using R version 3.2.0 (23). Coefficients of variation (CV) were calculated to express inter- and intra-individual variation. We applied the lme4-package (24) for linear mixed effects analysis. For the variables summarized on a daily basis (see Table 1), we first wanted to see if they were affected by the lameness status of the cows. Days in milk were included in this model because feeding behavior, in particular feed intake is known to change with days in milk. The resulting model is:

(M1) $$Y_{\mathrm{ijk}}=\mu +{\text{LAME}}_{i}+{\text{DIM}}_{j}+{\text{LAME}}_{i}\times {\text{DIM}}_{j}+{\text{cow}}_{k}+\varepsilon_{\mathrm{ijk}}$$

*Y* represents the variables summarized on a daily basis, μ was the overall mean, lameness (LAME; *i* = lame; not lame), days in milk (DIM, *j* = 54, …, 384 days), and their interaction term were fixed effects, cow (*k* = 1, …, 16) was random effect, and ε was the error term. If the interaction term was non-significant, the model was re-run without the interaction. Visual inspection of residual plots did not reveal any obvious deviations from homoscedasticity or normality. *P*-values were obtained by likelihood ratio test of the full model against the reduced model (*Y*_ijk_ = μ + cow_k_ + ε_ijk_). Significance was determined as *P* < 0.05.

Preliminary analysis of the variables summarized on a daily basis showed that the Pearson correlation coefficients between daily rumination time and feeding time (*r* = 0.22, *P* < 0.001) and between rumination time and milk yield were small (*r* = 0.31, *P* < 0.001). The highest correlation was between feeding time and feeding rate (*r* = −0.66, *P* < 0.001). Although daily rumination time was not found to be affected by lameness in M1, it could reasonably be expected to be affected by daily feeding characteristics (Table 1) and milk yield. Accordingly, this was assessed using the following model, which includes lameness because some of the feeding characteristics were influenced by this factor:

(M2) $$\begin{array}{l} {\text{dRUMtime}}_{\mathrm{ikl}}=\mu +{\text{LAME}}_{i}+{\text{MILK}}_{l}+\text{dFchar}\\ \;+{\text{LAME}}_{i}\times \text{dFchar}+{\mathrm{MILK}}_{\text{l}}\times \text{dFchar}\\ \;+{\text{LAME}}_{i}\times {\mathrm{MILK}}_{\text{l}}+{\text{LAME}}_{i}\times {\text{MILK}}_{l}\times \text{dFchar}+{\text{cow}}_{k}+\varepsilon_{\mathrm{ikl}} \end{array}$$

In M2, dFchar represents either daily feeding time, feeding rate, FMI or feeding frequency. Lameness (LAME; *i* = lame; not lame), daily milk yield (MILK; l = 4.9, …, 44.5 l/day), dFchar, and all their interactions were fixed effects. As random effects, we fitted cow (*k* = 1, …, 16), and ε was the error term. Models were reduced by omitting non-significant interactions one by one, starting with the three-way interaction. The residual sum of squares (RSS) was calculated for each model to enable calculation of *R*^2^, which we did using the RSS from a model only containing the intercept and cow as random effect, thereby expressing total variation in data the following way: *R*^2^ = (total variation − model variation)/total variation. For model comparison, we used a combination of *R*^2^, AIC, and BIC to decide which of the feeding characteristics yielded the best fitting model.

When looking at feeding behavior within day, i.e., at the level of feed station visits, it is well known that there is a relation between intake per visit (vFMI) and visit duration (vFdur). Thus, we tested whether the slope of this relationship was affected by lameness and also by milk yield, using the following model:

(M3) $$\begin{array}{l} {\text{vFMI}}_{\mathrm{ikm}}=a\times \text{vFdur}+b\times {\text{LAME}}_{i}\times \text{vFdur}\\ \;+c\times {\text{MILK}}_{m}\times \text{vFdur}+d\times {\text{cow}}_{k}\times \text{vFdur}+\varepsilon_{\mathrm{ikm}} \end{array}$$

where a, b, c, and d are slope coefficients. As fixed effects, we used vFdur (continuous variable), the interaction terms between vFdur and mean lameness score during the experiment (LAME; *j* = 1.25, …, 4.25) and between vFdur and mean milk yield during the experiment (MILK; *m* = 10.3, …, 37.0 l/day), respectively. No intercept was fitted as by definition zero vFdur can only have zero vFMI, this also allows us to test the slopes for significant differences due to lameness (interaction terms on vFdur). As random effects, we fitted the interaction between vFdur and cow (*k* = 1, …, 16) to allow for a random slope for the effect of vFdur for each cow, and ε was the error term.

## Results

### Inter- and Intra-Individual Variation

The inter-individual and intra-individual means, SD and CV for the feeding and rumination characteristics on a daily basis are reported by lameness category in Table 2. In general, the intra-individual variation was low, i.e., 5–15% for both lame and non-lame cows, whereas inter-individual variation was much higher, up to 50%. However, lameness did not affect feeding and rumination behaviors in the same way, because the inter-individual variation of the feeding characteristics was much higher in the lame cows (26–50%) than in non-lame cows (17–29%), contrary to the inter-individual variation of the rumination characteristics, which differed less regardless the lameness status (10–20%).

**Table 2:** **Inter-individual and intra-individual mean, SD, and coefficient of variation (CV, %) by lameness category for the feeding, rumination, and milk characteristics, *n* = 311 cow days, hereof 129 non-lame and 182 lame cow days**.

	Inter-individual						Intra-individual					

	Non-lame			Lame			Non-lame			Lame		
	Mean	SD	CV	Mean	SD	CV	Mean	SD	CV	mean	SD	CV
Milk yield, l/day^a^	21.4	8.85	41.4	24.7	5.96	24.1	19.4	2.82	14.5	24.6	1.35	5.5
FMI, kg/day	36.0	6.48	18.0	35.5	9.63	27.1	36.0	2.21	6.1	35.0	3.35	9.6
Feeding time, min/day	182	37.4	20.5	126	44.2	35.1	186	19.4	10.4	122	15.1	12.4
Feeding frequency, visits/day	57.9	10.1	17.4	35.1	17.8	50.1	58.0	5.60	9.7	35.2	3.17	9.0
Mean FMI, kg/visit	0.66	0.190	28.8	1.16	0.363	31.3	0.66	0.058	8.8	1.15	0.107	9.3
Mean feeding duration, s/visit	195	45.9	23.5	246	80.6	32.8	199	21.9	11.0	240	25.2	10.5
Daily feeding rate, g/min	206	48.1	23.3	289	74.4	25.7	203	14.2	7.0	294	17.1	5.8
Rumination time, min/day	490	73.6	15.0	478	46.4	9.7	482	25.2	5.2	480	25.1	5.2
Rumination frequency, events/day	24.5	4.79	19.6	25.0	4.26	17.0	24.4	2.75	11.3	25.0	2.56	10.2
Mean rumination duration, min/event	20.8	3.59	17.2	20.1	3.60	17.9	20.8	2.82	13.6	20.3	2.17	10.7

*^a^Milk yield based on 285 cow days, hereof 123 non-lame and 162 lame cow days*.

*FMI, fresh matter intake*.

### Feeding, Rumination, and Lameness Effects

The least square mean (LSM), SE, and *P*-values for the effect of lameness on daily feeding and rumination behavior sums derived from M1 are reported in Table 3. The interaction between lameness and DIM was insignificant for all variables with the exception of mean FMI per visit, so the results in the table are from the reduced model containing only the main effect of lameness. Lameness affected several of the feeding behaviors significantly but none of the rumination behaviors: lame cows made 46% less visits to the feed stations and fed for 44% shorter per day than non-lame cows. Contrastingly, lame cows ate 40% faster than their non-lame pen-mates. A significant interaction between lameness and DIM meant that lame cows in early lactation had a larger mean FMI per visit than non-lame cows (−0.004 kg/visit per day, SE = 0.0014, *P* = 0.006), but this difference between lame and non-lame cows in mid to late lactation diminished. Feeding time increased significantly with DIM (0.32 min/day, SE = 0.129, *P* = 0.03). Cows ruminated 481 min/day with a frequency of about 25 events/day, both unaffected by lameness and DIM. Daily milk yield and daily FMI did not differ significantly between lame and non-lame cows, but as expected, milk yield decreased significantly with DIM (−0.05 l/day, SE = 0.015, *P* < 0.001), and FMI increased significantly with DIM (0.07 kg/day, SE = 0.026, *P* = 0.01).

**Table 3:** **Least square means (LSM), SE, and *P*-values for daily feeding, rumination, and milk yield characteristics of the effect of lameness derived from model 1 (M1)**.

Characteristic	Non-lame		Lame		*P*
	LSM	SE	LSM	SE	
Milk yield, l/day^a^	18.8	2.84	25.9	2.44	n.s.
FMI, kg/day	39.4	3.96	33.8	3.36	n.s.
Feeding time, min/day	197	20.3	119	17.2	0.004
Feeding frequency, visits/day	60.3	6.29	33.9	5.34	0.002
Rumination time, min/day	480	25.1	482	21.2	n.s.
Rumination frequency, events/day	23.4	1.39	25.6	1.17	n.s.
Mean feeding duration, s/visit	201	28.8	243	24.3	n.s.
Daily feeding rate, g/min	206	26.2	289	22.4	0.02

*Cows with a locomotion score ≥3 were defined as lame, *n* = 311 cow days, hereof 129 non-lame and 182 lame cow days*.

*^a^Milk yield based on 285 cow days, hereof 123 non-lame and 162 lame cow days*.

*FMI, fresh matter intake*.

**Figure 1:**
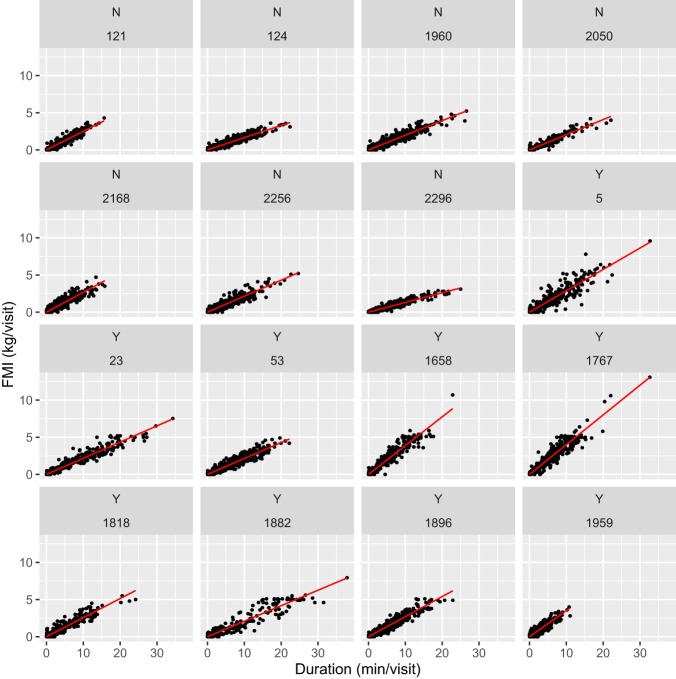
**Fresh matter intake (FMI, kilogram per visit) relative to visit duration (minute per visit) by cow with lameness category (lame: *Y*; non-lame: *N*) depicted before cow ID, *n* = 13,908 visits**. Overall feeding rate is the slope of the regression line.

In Figure 1, FMI per visit is plotted against feeding duration per visit for each cow. The overall feeding rate for each cow (cFR) is the slope of the regression line, revealing different cFR across cows. The five fastest feeding cows were lame; the three slowest feeding cows were non-lame, whereas the individuals feeding at an intermediate rate were a mixture of lame and non-lame cows. The larger mean FMI per visit in the lame cows manifests itself by more visits close to the limit of 5 kg/visit imposed electronically on the feed stations.

**Figure 2:**
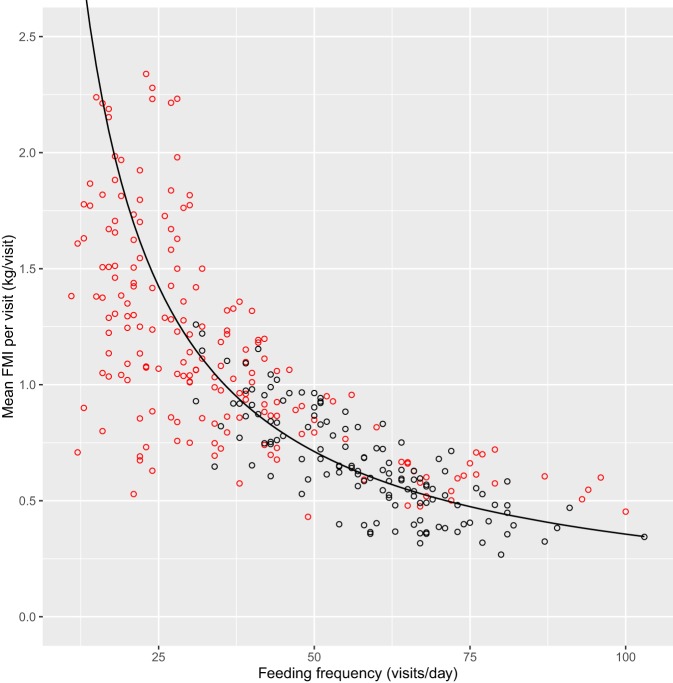
**Mean fresh matter intake (FMI) per visit (kilogram per visit) relative to feeding frequency (visits/day) grouped by lameness category (lame: red; non-lame: black) with a line indicating the mean FMI of 35.6 kg/day, *n* = 311 cow days**.

**Figure 3:**
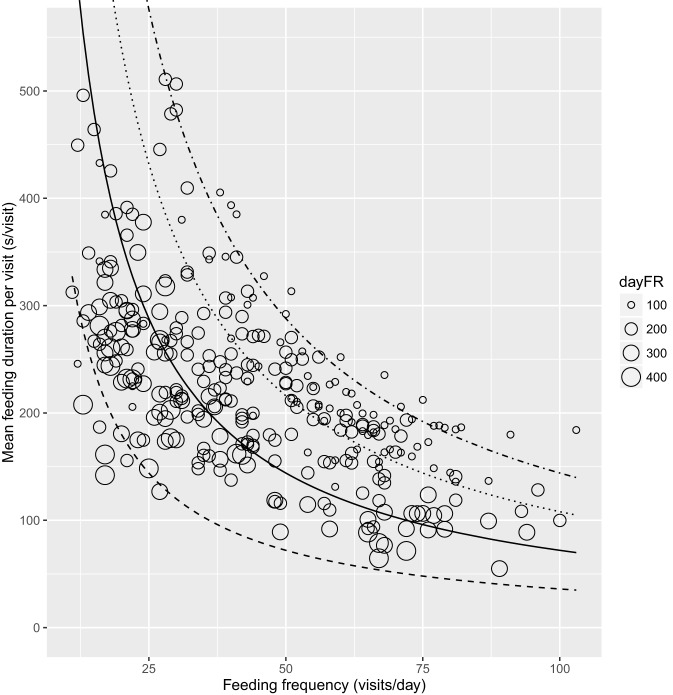
**Mean feeding duration per visit (second per visit) relative to feeding frequency (visits per day) grouped by daily feeding rate (dayFR, gram per minute) with lines representing constant feeding times of 1 (dashed), 2 (solid), 3 (dotted), and 4 h/day (dot-dashed), *n* = 311 cow days**.

The association between mean FMI per visit and feeding frequency grouped by lameness category is shown in Figure 2 with a line indicating the overall mean FMI of 35.6 kg/day. The fewer feeder visits with higher intakes for the lame cows is evident in the clustering of the data points toward the upper left-hand part of the FMI line (Figure 2). Figure 3 shows mean feeding duration per feeding frequency grouped by daily feeding rate with the lines indicating constant feeding times of 1, 2, 3, and 4 h/day.

Based on the 13,908 feed station visits, M3 explored how the relationship between duration (vFdur) and intake per visit (vFMI), which is the feeding rate, was affected by locomotion score and milk yield, both of which were significant. Thus, the feeding rate estimate was 119 g/min (SE = 3.95, *P* < 0.001). Feeding rate increased a highly significant 36 g/min per increasing locomotion score unit (SE = 1.05, *P* < 0.001), whereas the increase of 0.32 g/min/l milk (SE = 0.16, *P* = 0.04) was much smaller yet significant.

### Associations between Feeding and Rumination

**Figure 4:**
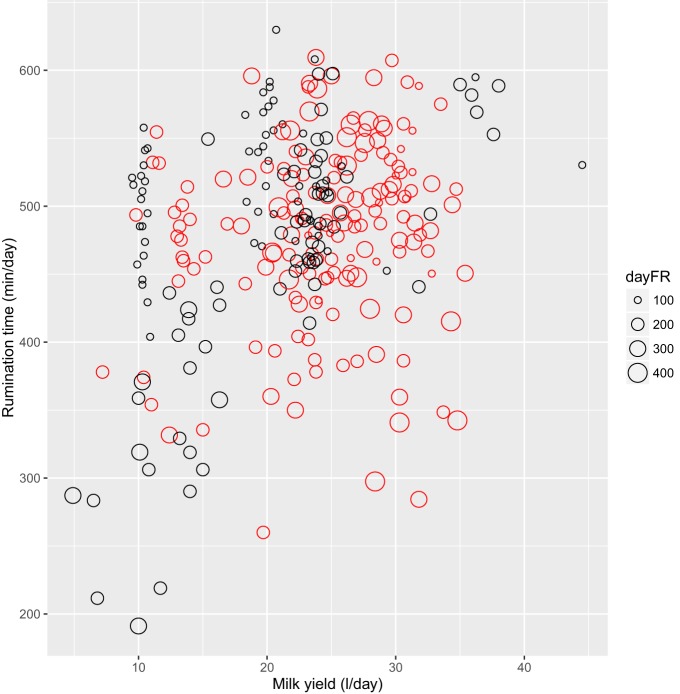
**Rumination time (minute per day) relative to milk yield (liter per day) grouped by daily feeding rate (dayFR, gram per minute) and lameness category (lame: red; non-lame: black), *n* = 285 cow days**.

The results of the model (M2) testing which aimed to explain the variation in daily rumination time by one of four feeding behavior characteristics, lameness, and milk yield are presented in Table 4, where the model name, RSS, *R*^2^, AIC, and BIC are reported. All models with more than one main effect were tested with interactions and with the exception of FMI; they could be reduced to contain only the main effects. In general, AIC and BIC were similar across all models, however, the models containing all three explanatory variables, i.e., milk yield, lameness, and one of the feeding behavior characteristics achieved the highest *R*^2^ and lowest RSS and AIC. Thus, an increase in *R*^2^ from 13 to 37% was achieved but at a relatively high cost in terms of additional explanatory variables. Of the models containing three main effects and no interactions, the models with feeding rate (M2.3.2) and feeding frequency (M2.3.3) performed equally well. In the model with feeding rate (M2.3.2), daily rumination time of 461 min/day (SE = 36.9) decreased slightly but significantly with increasing feeding rate (-0.24 min/day, SE = 0.09, *P* = 0.009), increased with increasing milk yield (3.57 min/day, SE = 1.10, *P* = 0.001), but was not affected significantly by lameness (−1.66 min/day, SE = 27.79, *P* > 0.05). Figure 4 depicts rumination time per day relative to feeding rate per day grouped by lameness and daily milk yield. With feeding frequency as explanatory variable (M2.3.3), the daily rumination time of 457 min/day (SE = 37.8) decreased with increasing feeding frequency (−0.83 min/day, SE = 0.31, *P* = 0.009); again milk yield increased rumination time significantly (3.71 min/day, SE = 1.14, *P* = 0.001), whereas lameness did not affect rumination (−40.9 min/day, SE = 30.9, *P* > 0.05). Rumination time (M2.4, 388 min/day, SE = 47.2) was affected by an interaction between lameness and FMI (−2.30 min/day, SE = 1.14, *P* = 0.04), meaning that non-lame cows increased their daily rumination time with increasing daily FMI, whereas lame cows decreased their rumination time with increasing FMI. Feeding time (M2.3.1) did not affect rumination time significantly (not reported).

**Table 4:** **Name, residual sum of squares (RSS), *R*^2^, Akaike’s information Criterion (AIC), and Bayesian information criterion (BIC) values for each model tested, ordered by decreasing RSS as derived from model M2, *n* = 285 cow days**.

Model	Name	RSS	*R*^2^	AIC	BIC
1 + cow	M2.0	488	–	3,111	3,122
One main effect					
LAME + cow	M2.1.1	423	0.133	3,113	3,127
dFtime + cow	M2.1.2	423	0.132	3,112	3,127
dFMI + cow	M2.1.3	419	0.142	3,108	3,122
dFR + cow	M2.1.4	418	0.143	3,105	3,120
dMY + cow	M2.1.5	418	0.144	3,102	3,117
dFfreq + cow	M2.1.6	416	0.147	3,107	3,121
Two main effects					
dMY + LAME + cow	M2.2.1	373	0.235	3,103	3,122
dMY + dFR + cow	M2.2.2	370	0.242	3,096	3,115
Three main effects					
dMY + LAME + dFtime + cow	M2.3.1	341	0.301	3,105	3,127
dMY + LAME + dFR + cow	M2.3.2	337	0.308	3,098	3,120
dMY + LAME + dFfreq + cow	M2.3.3	335	0.313	3,099	3,121
Three main effects and interaction					
dMY + LAME + dFMI + LAME × dFMI + cow	M2.4	310	0.365	3,098	3,124

*In all models, rumination time is the response variable. See Table 1 for explanation of abbreviations*.

## Discussion

This study aimed to characterize the individual feeding and ruminating behavior of dairy cows obtained from automatically and simultaneously registered sensor data, and use this to quantify the effects of lameness on feeding and rumination. These aspects will be discussed in turn below.

### Can Cows Be Characterized by Their Feeding and Rumination Behavior?

In order to characterize an animal, we need measures that vary little within animals but have a large variation between animals. In our study, within-cow variation was between 5 and 15% for all behavior characteristics measured, compared to a variation of 10–50% between cows. Feeding behavior in lame cows varied considerably more compared with the feeding behavior of non-lame cows, but interestingly, lameness did not affect the rumination behaviors to the same extent. Overall, rumination behavior varied less between individuals than feeding behavior, in agreement with a recent study (25). Among the behavioral characteristics, rumination time and feeding rate were the most stable aspects within cows. Thus, cows can indeed be characterized by their feeding and rumination behavior. Furthermore, feeding and rumination measures do have the potential to provide good phenotypic characteristics of group housed dairy cows.

In the present study, the three best descriptors mentioned above were correlated, i.e., rumination time decreased with increasing feeding rate and increased with increasing milk yield. It is possible that a higher feeding rate leads to a change in rumination efficiency, such that cows chew faster, too. Measuring this would require a more detailed quantification of rumination by measuring the number and – ideally – weight of boluses swallowed over time. Moreover, cows feeding on TMR are known to sort through the food (6), and it is possible that lame cows change their sorting behavior in order to minimize the time spent feeding, which may have a knock-on effect on rumination time. However, measuring sorting behavior was outside the scope of this study.

This study showed that daily feeding time did not affect daily rumination time significantly, which is in agreement with another study (26). However, periods of feeding are expected to be followed by periods of rumination (13), and it is likely that the use of daily values in the present study is disguising ultradian rhythms. This is supported by Schirmann et al. (26), who found a correlation between feeding time and rumination time by imposing a 4-h lag between 2-h periods of measurement of these two variables. Short-term reductions in feeding time and rumination time have been found for cows in estrus, which ruminated on average 75 min less and fed for 58 min less on the day of artificial insemination compared with 3–7 days prior to insemination (16). Some of the cows may have been in estrus, which would have lasted 14.1 ± 4.5 h (27) thereby only affecting the feeding and rumination pattern for a fraction of the 24-day period, therefore reproductive status was not taken into consideration in the present study.

The ranges of feeding and rumination variables found in the present study are comparable to others reported. However, the fresh feed intake of 35.6 kg/day in the present study was lower than that found by others using similar feed station systems (19, 28). This may be due to most of our cows being in late lactation – and therefore yielding considerably less – and to differences in TMR compositions. The non-lame cows of our study fed for 197 min/day, which was also within the range from 104 to 264 min/day reported by others (10, 11, 16). Here it should be noted, that Norring and colleagues (10), who reported a feeding time of 104 min/day excluded visits shorter than 1 min from analysis, thereby introducing an underestimation of duration. The feeding rate of the non-lame cows in our study of 206 g/min was within the range of the feeding rates of 159 to 340 g/min reported for healthy cows in other studies (10, 19, 28). The cows of our study ruminated 481 min/day, which was within the range from 389 to 509 min/day reported by others (11, 16). We found no effect of DIM on daily rumination time, in contrast to Miguel-Pacheco and colleagues (11), who found cows greater than 130 DIM ruminated more compared with those less than 130 DIM, whereas in another study, rumination bouts decreased with DIM (29). After parturition dwarf goats would double both their daily food intake and feeding rate, but total rumination time was lower despite the massive increase in intake (30).

As the cows were kept in one group, their behavior would have been influenced by their pen mates. Subordinate cows have been found to eat more quickly than their dominant conspecifics when fed in a social context (31). In the present study, no measures of social hierarchy were made, and the competition for feeder space with 1.7 cows/feed station was intermediate of the 1–2 cows/feeder space most frequently used in the literature [e.g., 1.1 (26) and 2.0 (16)]. The group was kept stable, as no new cows were introduced during the experimental period of our study.

### Effects of Lameness on Feeding and Rumination Behavior

We found that lame cows differed significantly from non-lame cows in three aspects of their feeding behavior, i.e., feeding time, frequency and rate. Lameness reduced daily feeding time by 46%, which agrees with the finding by Miguel-Pacheco and colleagues (11), whereas others have reported a less profound decrease of 13% (10). Lameness also reduced feeding frequency by 44% in our study, again similar to that found by Miguel-Pacheco and colleagues (11). Gonzàlez and colleagues reported a decline of 0.35 visits/day during the month preceding a lameness diagnosis. The reduced feeding frequency and feeding time found in our study did not affect the daily FMI of the lame cows, in agreement with Gonzàlez et al. (9). This was because the lame cows in our study appeared to compensate by having a 40% faster rate of eating. Increased feeding rates in lame cows compared to non-lame cows have also been found by others, although to a lesser extent [11% (10) and 21% (9)].

Constrained feeding time makes cows eat faster (32), and in this respect, lameness may be conceived as a feeding time constraint by cows. In our study, the feeding rate increased more in lame cows than in non-lame cows when FMI increased, thereby indicating that the lameness constraint on feeding time forces cows to eat quicker, a situation that (in the context of overcrowding) is known to be an indicator of stress (15). Other types of illness have been found to affect feeding behavior differently. For example, cows subjected to repeated ruminal acidosis challenges ruminated less, increased their feeding time and ate slower (6). These differences between effects of acidosis and lameness may enable us to detect and to distinguish between lameness and acidosis based on PLF technologies.

Rumination is essential to proper rumen function. Rumination is often done while lying down (26), and with 24 cubicles available for 20 cows in our study, rumination while lying down was not constrained for the cows studied. Concurring with another study (33), we found that lameness did not affect daily rumination time. Small but significant effects of lameness on rumination have, however, been reported. For instance, the 7-day average of the night-period rumination time was 13 min shorter for lame cows than non-lame cows (17), moreover, rumination time was reduced by 8 min/day in the two days following a lame locomotion score (11).

### Future Perspectives and Conclusions

Our results indicate that, when compared to not lame cows, lame cows are likely to exhibit a different feeding behavior such as increased feeding rate and decreased feeding time, whereas rumination time seems much less affected by lameness. This is to our knowledge a new finding. Therefore, if farmers focus solely on measuring rumination automatically, they may not detect lameness problems, although automated rumination monitoring may be very useful for detecting other diseases that do change rumination time, like ruminal acidosis. Lameness problems would require measurement of feeding as well as rumination to be noticed automatically. Moreover, using a combination of two or more feeding behaviors is likely to increase the accuracy of detecting problems.

Until now, the relationship between feeding behavior measures and state of lameness has not been explored as a means to phenotype lameness continuously, which can be used in the genetic selection against propensity to become lame. Accelerometer data are of particular interest in this context, because in addition to providing information of lameness on-farm (34), they also provide an automated means to access additional behavioral data, like shown for rumination in the present study. We found that rumination showed more variation between cows than within cows, but that feeding variation was up to five times higher between cows than within cows, meaning that feeding and rumination characteristics have the potential to provide good phenotypic measures of ruminal robustness. In addition, high daily feeding rate, a low number of visits to feeders, and shorter time spent feeding possibly combined with an increased variation in feeding behavior observed between cows, may serve as lameness indicators.

The present study compared lame and non-lame individuals using PLF systems. The utilization of PLF systems will enable real-time monitoring of within-cow changes in feeding behavior using shorter time-frames (ultradian) and incorporating the natural cyclicity of feeding. Future research should seek to further quantify the changes in feeding behavior associated with changes in locomotion score to enable lameness detection at the individual level. This would open the door to go a step further and move from locomotion scores to a characterization of lameness on a continuous “degree of lameness” scale. The relationship between feeding rate, milk yield, and rumination time found here should be verified in a study with more cows and a more complete set of feed station records. Also, the ability to characterize rumination in more detail, such as quantifying the number and weight of boluses swallowed over time, would add valuable information for use in the phenotyping of dairy cows.

## Ethical Note

This study was carried out in accordance with the recommendations of the SRUC Code of Practice, and the protocol was approved by the Animal Ethic Committee of SRUC.

## Author Contributions

VT: made substantial contributions to conception of the work, data acquisition, analysis, and interpretation, drafted and revised the work, and wrote the final version. BN: made substantial contributions to data analysis and interpretation and drafted and revised the work. P-ER and SG-R: made substantial contributions to data analysis and interpretation and revised the work. JK and CM: made substantial contributions to data acquisition and analysis and drafted the work. NF: made substantial contributions to conception of the work and data analysis and revised the work. All authors approved the final version of the paper and agree to be accountable for all aspects of the work.

## Conflict of Interest Statement

The authors declare that the research was conducted in the absence of any commercial or financial relationships that could be construed as a potential conflict of interest.
